# South African paramedic perspectives on prehospital palliative care

**DOI:** 10.1186/s12904-020-00663-5

**Published:** 2020-10-08

**Authors:** Caleb Hanson Gage, Heike Geduld, Willem Stassen

**Affiliations:** 1 Division of Emergency Medicine, University of Cape Town, Cape Town, 7935 South Africa; 2 Division of Emergency Medicine, Stellenbosch University, Cape Town, South Africa

**Keywords:** Palliative, End-of-life, Prehospital, EMS providers, Paramedic

## Abstract

**Background:**

Palliative care is typically performed in-hospital. However, Emergency Medical Service (EMS) providers are uniquely positioned to deliver early palliative care as they are often the first point of medical contact. The aim of this study was to gather the perspectives of advanced life support (ALS) providers within the South African private EMS sector regarding pre-hospital palliative care in terms of its importance, feasibility and barriers to its practice.

**Methods:**

A qualitative study design employing semi-structured one-on-one interviews was used. Six interviews with experienced, higher education qualified, South African ALS providers were conducted. Content analysis, with an inductive-dominant approach, was performed to identify categories within verbatim transcripts of the interview audio-recordings.

**Results:**

Four categories arose from analysis of six interviews: 1) need for pre-hospital palliative care, 2) function of pre-hospital healthcare providers concerning palliative care, 3) challenges to pre-hospital palliative care and 4) ideas for implementing pre-hospital palliative care. According to the interviewees of this study, pre-hospital palliative care in South Africa is needed and EMS providers can play a valuable role, however, many challenges such as a lack of education and EMS system and mindset barriers exist.

**Conclusion:**

Challenges to pre-hospital palliative care may be overcome by development of guidelines, training, and a multi-disciplinary approach to pre-hospital palliative care.

## Background

The World Health Organisation (WHO) defines palliative care as *‘an approach that improves the quality of life of patients and their families facing the problem associated with life-threatening illness, through the prevention and relief of suffering by means of early identification and impeccable assessment and treatment of pain and other problems, physical, psychosocial and spiritual.’* [1] This includes a wide variety of situations such as chronic illness and end-of-life care [2].

Such treatment is typically performed by palliative care specialists who work in-hospital. However, emergency medical services (EMS) often encounter patients requiring palliative care as these patients may have acute exacerbations of illness, progress towards end-of-life or require transport to a medical facility [3–9]. As the first point of medical contact, EMS providers are thus uniquely positioned to deliver early palliative care in the home [10]. This has great potential benefit for patient comfort, early identification and relief of suffering and earlier referral to hospice care [10, 11].

Despite this unique position there is an overall lack of guidance within EMS systems to manage palliative patients [5, 6, 10]. In the United States of America (USA) only 5–6% of EMS systems have protocols for palliative care [6, 10]. In addition, there is no specific pre-hospital emergency care curricula on the subject, resulting in a lack of education and training for EMS providers [3–5, 12, 13]. This might stem from the historical focus of EMS training which primarily involves immediate measures to preserve life or limb until definitive care is reached [11]. This focus has resulted in an EMS ethos of ‘saving lives.’ [5, 12] Palliative care, on the other hand, is not focussed on ‘saving lives’, but rather the prevention and relief of suffering [1]. Therefore, palliative care may seem to conflict with emergency care, placing EMS providers in difficult situations when confronted with palliative care patients [8, 12, 14].

The Sub-Saharan African region faces the world’s most significant health crisis [15]. South Africa itself faces what has been termed a “quadruple burden of disease” due to communicable diseases such as human immunodeficiency virus (HIV), acquired immunodeficiency syndrome (AIDS), high maternal and paediatric mortality rates, non-communicable disease as well as injury [16]. The large number of patients suffering from these diseases and the life-limiting complications thereof, results in increased need for palliative care in the country as noted by the South African Minister of Health [17].

Access to health care for patients suffering from these diseases is a further challenge in the Sub-Saharan African setting [15, 18, 19]. In South Africa, EMS are often contacted by those without access to transport to provide this service. Thus, South African EMS providers may frequently encounter not only high acuity emergency patients, but many ill HIV/AIDS, cancer and other chronically ill patients requiring palliative care who are unable to access healthcare via alternative means [20]. European studies have found that approximately 3–5% of all pre-hospital calls involve palliative care situations [2, 21, 22]. With the quadruple burden of disease and limited access in the South African setting, this percentage is likely higher as these factors result in increased frequency of contact between EMS providers and patients requiring palliative care.

The aim of this study was to gather the perspectives of advanced life support (ALS) providers within the South African private EMS sector regarding pre-hospital palliative care in terms of its importance, feasibility and barriers to its practice. To our knowledge, no research has been produced in the (South) African setting regarding prehospital palliative care.

## Methods

### Design

A qualitative study design employing individual semi-structured interviews was used. The theoretical orientation underpinning this study was descriptive phenomenology as it sought to describe perspectives of EMS providers regarding palliative care [23].

### Setting

Each interview was held in a private setting agreed upon by the primary researcher and participant. To avoid interruptions, interviews were held with off-duty ALS providers.

ALS providers were chosen as the population of the study due to their broad scope of practice when compared to other cadres of provider. This scope of practice, although intended for emergency patients, includes the ability to perform certain palliative care interventions such as opioid administration.

The private South African EMS sector was chosen as the research team had greater access to the ALS providers within the private system. The private system was more suited to the inclusion and exclusion criteria of the study as it contained a greater number of ALS providers with higher education (HE) qualifications. For the purposes of this study the “private EMS sector” refers to non-governmental, for-profit EMS companies.

To provide context, South Africa has two separate healthcare systems: private and state [24]. State healthcare is provided by the South African government to all citizens while private healthcare is accessible only to those with the ability to pay for services or those with healthcare insurance. The South African EMS sector, in both private and state systems, has a three-tiered “level of care” model; basic, intermediate and advanced life support (BLS, ILS, ALS) [25]. In this manner, prehospital emergency care is paramedic rather than physician led.

### Data collection

The interview schedule contained four questions referencing career background, importance of, concerns with, and opinions on palliative care feasibility in the South African pre-hospital environment. Pre-determined prompts and probes were included for each question to enhance data collection by thoroughly exploring the interviewees’ perspectives. For example, when inquiring concerning the perceived importance of prehospital palliative care, the following probes/prompts were used to stimulate further discussion: *Is there opportunity to provide this care? How many of your patients require this care?* The interview schedule was jointly developed by Caleb Gage (CG) and Willem Stassen (WS), specifically for the study, based on the contextualisation of the limited available literature. The interview schedule developed for this study is available as Additional file 1.

Interviews were performed by the primary investigator, who was trained beforehand in qualitative interviewing. Prior to data collection a pilot interview was performed between the primary investigator and supervisor to ensure consistency and quality of interview technique. Data from this interview was not included in the final analysis.

Six interviews were held from March to May 2018. Interviews lasted approximately 20–35 min. All six interviewees knew the interviewer from a mutual training institution. Participants were aware the study was part of the interviewer’s post-graduate studies.

ALS providers were invited to participate in the study via email circulation within a local database of South African paramedics who have volunteered their names to be a part of the database. Three ALS providers indicated their willingness to participate and a further three providers were selected on a convenience basis.

Inclusion and exclusion criteria were as follows: Inclusion - Qualified ALS providers with HE qualifications (National Diploma, Bachelor of Technology and Bachelor of Health Sciences in Emergency Medical Care), currently working in, or with recent previous experience in, the South African private EMS sector with a minimum of 2 years private sector operational experience. Recent previous experience referred to South African private sector operational experience, within the last 3 years if the ALS provider was not currently working in the private sector. Exclusion - ALS providers without pre-hospital, operational experience in the South African private EMS sector.

### Analysis

Interview recordings, created with a recording application on a cellular device, were manually transcribed verbatim by CG. Transcriptions were identified by interview number and gender (e.g. Interview #1 M).

**Fig. 1:**
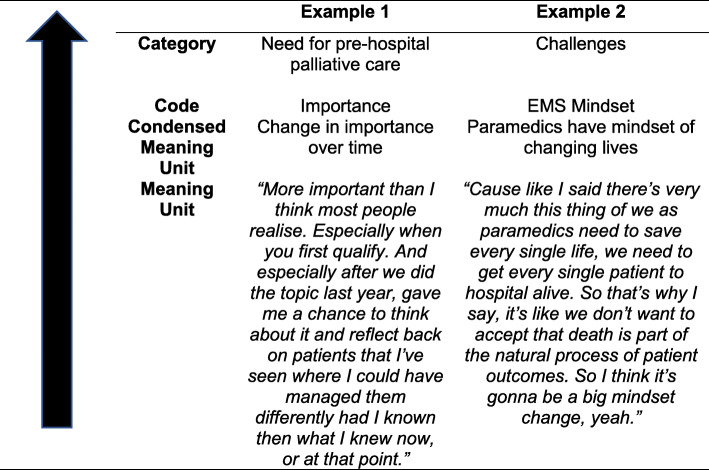
Categorical development during analysis from lower to higher levels of abstraction

Content analysis of transcribed data was performed using the framework of Braun and Clark [26, 27]. These techniques were conducted alongside data collection as field notes were made during interviews. To become further familiar with the data, an initial ‘floating reading’ was done. Analysis of the transcribed data was then performed using an inductive-dominant approach. Meaning units were captured, condensed, coded and categorized (see Fig. 1) using NVivo version 12 software [28]. Data coding was performed by the interviewer alone. Researcher triangulation was performed between CG and WS where categories and codes were discussed, refined and finalised. Member checking of these categories and codes was performed hereafter.

Data saturation, as detailed by Saunders, et al. was reached after six interviews [29]. This was evidenced by repetitive comments by interviewees and lack of additional data being gained after the sixth interview. Details of the interviewees and interviewer are presented in Table 1.

**Table 1:** Demographic characteristics of interviewees and interviewer

	Gender	Qualification	Post-Grad Experience	Areas of Experience
Interviewee #1	Male	ECP ^a^	4 years	Operations: Road, HEMS ^b^, Fixed Wing
Interviewee #2	Male	ECP	3 years	Operations: Road, HEMS, Fixed Wing Other: Psychology
Interviewee #3	Female	ECP	3 years	Operations: Road, HEMS, Fixed Wing, ICU ^c^ Ambulance
Interviewee #4	Female	ECP	6 years	Operations: Road, HEMS, Fixed Wing, ICU Ambulance Other: Nursing, Lecturing
Interviewee #5	Male	ECP	2 years	Operations: Road, ICU Ambulance
Interviewee #6	Female	ECP	3 years	Operations: Road, HEMS, ICU Ambulance
Interviewer (CG)	Male	ECP	5 years	Operations: Road, HEMS, Fixed Wing Other: Contract, Primary Health

^a^ *ECP* Emergency Care Practitioner. This equates to a 4-year bachelor’s degree in Emergency Medical Care, ^b^ *HEMS* Helicopter Emergency Service, ^c^ *ICU* Intensive Care Unit

### Reflexivity

I (CG) carried out the interviews and performed this study as part of the Master’s degree in Emergency Medicine at the University of Cape Town (UCT). During the study, I held positions as a paramedic working in primary healthcare in West Africa and as a helicopter EMS paramedic for a private company in South Africa. As I had no prior experience in qualitative research, I completed an online training course in qualitative research methodology before commencement of the study [30]. During the interviews I recognised my own personal perspectives (i.e. palliative care *should* be performed in the South African pre-hospital setting and *would* be useful) and actively avoided these by refraining from asking leading questions and adhering closely to the discussion schedule. While several interviewees appeared to agree with my perspectives, this was not unanimous as at least one interviewee identified other “*more important*” priorities. Throughout the process I felt my interview technique improved. I also felt the discussion schedule was logical as answers to the pre-determined questions flowed naturally into the next questions during the interviews.

### Trustworthiness

To ensure trustworthiness of the data the framework of Guba was used in seeking credibility, transferability, dependability and confirmability [31]. The following steps were taken to ensure these criteria in the study [31]:

- Credibility: Interview technique was practiced with a pilot interview. Participants were given the opportunity to refuse participation and participated voluntarily. During data collection and analysis frequent communication and debriefing sessions were held between the interviewer and study supervisors. Researcher triangulation was carried out and member checking was performed. A reflexive commentary is provided.
- Transferability: A thorough description of data collection methods is given. Interview duration and the data collection period are reported. Study results are further discussed in relation to available literature.
- Dependability: Full descriptions of research design and implementation, a reflexive commentary as well as the details of data gathering are presented.
- Confirmability: Preliminary perspectives held by the research team which are not demonstrated in the data are discussed and reflected upon in the reflexivity section. Researcher triangulation and member checking were performed. In addition, verbatim transcriptions of the audio data were produced.

Ethical approval for the study was provided by the University of Cape Town’s Human Research Ethics Committee (HREC Reference Number: 058/2018). This manuscript complies with the COnsolidated Criteria for Reporting Qualitative (COREQ) research checklist [32].

## Results

Six participants were interviewed (Table 1). Interviews lasted between 20 and 35 min (average: 30 min) Four categories, with their subcategories, emerged from these interviews as detailed in Table 2. According to the interviewees of this study, pre-hospital palliative care in South Africa is needed and EMS providers can play a valuable role, however, many challenges exist.

**Table 2:** Categories and subcategories emerging from participant interviews

**1) Need for pre-hospital palliative care**	
a) opportunity, neglect and importance b) ethics, patient rights and human dignity	
**2) Function of EMS providers concerning palliative care**	
a) facilitation of care b) improving quality of life c) home-based care	
**3) Challenges to pre-hospital palliative care**	
a) EMS mindset b) lack of training c) resource and scope of practice considerations d) EMS system failures e) difficulties specific to the pre-hospital field	
**4) Ideas for implementing pre-hospital palliative care**	
a) creation of guidelines b) multi-disciplinary approach c) creation of pre-hospital palliative care specialists/teams	

## Discussion

Participants were asked to share their perceptions regarding importance of, concerns with, and opinions on palliative care feasibility in the South African pre-hospital environment (See Additional file 1). Below, each category which emerged is discussed with supporting quotations extracted from the interviews.

### Need for pre-hospital palliative care

Based on their experiences all participants stated there is a need for pre-hospital palliative care in South Africa. Reasons given were a) the opportunities to provide palliative care, neglect and importance of palliative care and b) ethics, patient rights and human dignity.

*a) Opportunity, neglect and importance.*

*“This is a huge, huge population we actually service that falls into that category: end-stage cancer, end-stage RVD* [retroviral disease – HIV/AIDS] *…*” – Interview #5 M.

“*… I think it’s actually more important than we realise.” – Interview #1 M.*

*“Opportunity* [to perform palliation] *yes, but I never took it.”* – Interviewee #6.

All interviewees detailed personal experiences regarding the opportunities they had to treat patients requiring palliative care. Specific populations of patients mentioned were those with terminal cancer, chronic mental and physical illnesses and the elderly. Several studies in High Income Countries (HICs) have noted similar palliative care patient populations to which EMS are called [5, 21, 33]. While these populations are served by South African EMS the opportunity to provide palliative care was seen to be neglected.

Participants detailed how experience played a role in their perception of pre-hospital palliative care’s importance. While early in their careers palliative care was not a focus, it became increasingly important with experience. Interviewees described palliative care in the South African pre-hospital setting as *“very”* and *“extremely important”.*

*“… especially after we did the topic* [palliative care] *last year, gave me a chance to think about it and reflect back on patients that I’ve seen where I could have managed them differently had I known then what I know now …”* – Interview #3F.

Wiese, et al. have shown in several studies involving pre-hospital palliative care that EMS provider experience plays an important role in palliative situation decision-making [9, 21, 34]. They found that experienced EMS providers were both more comfortable and more confident in managing palliative situations [9, 21, 34].

*b) Ethics, patient rights and human dignity.*

One participant stated that ethically *“we owe it”* to patients to provide relief from suffering. This includes the provision of palliative care. Another interviewee mentioned the importance of treating end-of-life patients in a dignified manner by avoiding overtreatment.

*“… if I put this patient in the ambulance what’s the minimum I can do to still preserve dignity …*? *”* – Interview #5 M.

The concepts of dignity and autonomy are patient rights and integral to palliative care [3, 9]. These rights, however, may not always coincide with EMS therapeutic goals [9]. Thus, in the prehospital setting it is important to take these patient rights into account particularly when managing palliative patients [3]. Current literature suggests this can be attained through integration of emergency and palliative healthcare; something the interviewees of this study recognised as a need in South Africa [7, 9, 35].

Another interviewee, taking the patient’s family into account, highlighted the danger of creating false hope in the case of overtreatment. This view is commensurate with the WHO position on palliative care [1]. Previous studies in which EMS providers have been interviewed concerning prehospital palliative care have demonstrated the consideration for family dynamics as an important part of managing palliative situations [7, 12].

For the above reasons, participants felt there is a need for palliative care in the South African prehospital setting. While some felt it was *“appropriate in the South African setting”* and *“should be implemented”,* others felt that despite its importance, other shortcomings within the South African EMS system should be addressed first. These included areas such as primary health care provision, system development and improvement in current resuscitative practices. Likewise, in European studies, while many EMS providers saw a need for developing prehospital palliative care, some did not consider this a priority given other pressing issues [8, 9, 21]. Further study is needed in the (South) African environment to identify top priorities and set a prehospital development agenda.

### Function of EMS providers concerning palliative care

Participants felt that EMS providers have an important role to play in providing palliative care. As Wiese, et al. stated, *“… so long as there is no extensive outpatient care for palliative care patients, emergency medical teams must continue to play an important role …”* [21] This includes the South African setting where both inpatient and outpatient palliative care is lacking [17]. According to participants, these roles are a) facilitation of care (including initiation of care, facilitation of further care and on-scene decision-making), b) improving quality of life and c) home-based care. Interviewees saw these roles as beneficial to both patient and family.

*a) Facilitation of care.*

*“What you do then determines what is going to happen for the rest of their course … So specifically pre-hospitally it’s such a big deal …”* – Interview #3F.

*“… that liaising, that mediator between the two* [treating doctor and patient] *is, I think, one of the most important functions we have in those kinds of situations with palliation.”* – Interview #2 M.

These facilitative roles suggested by participants take advantage of the unique position of EMS as the first point of medical contact for pre-hospital patients requiring palliative care. EMS providers can assess the patient, *“take a step back”* and communicate with the patient and family members as to their desires for treatment and be a part of the decision-making process. As commonly described elsewhere, this initial pre-hospital decision-making sets the trajectory for subsequent palliative patient care [5, 10, 13]. In the German setting, it has been found that poor decision-making, by EMS providers inexperienced in palliative care, results in in-hospital treatments which are not desired by the patient or the patient’s family [21].

Importantly one practitioner pointed out that while EMS providers can and should be a part of this decision-making process, it is not their place to make palliative care decisions in isolation.

*“We can be part of the system that makes that decision, that executes the decision, but I don’t feel it’s our place to make that decision.”* – Interview #4F.

A Swiss prehospital palliative care study supports this viewpoint; the authors calling for collaboration between palliative care systems and EMS [4]. Likewise, an investigation, in which EMS providers were interviewed concerning palliative care emergencies, highlighted the desirable nature of a stronger integration between palliative and EMS systems [9].

*b) Improving quality of life.*

Interviewees identified improving quality of life as part of their role in palliation, consistent with the WHO definition of palliative care [1]. Describing an end-of-life situation where a baby has undergone palliative care and is at home with family, Interviewee #4F said, *“To be picked up, wrapped in a blanket and held by your mom until you pass away vs. lying in a hospital crib connected to vents and monitoring … you improve , in my opinion, the quality of life at the end because you can’t improve quality of life anymore with medical support … And then you also improve the quality of life for the family cause they get to have that experience.”* Thus, EMS providers could facilitate improved quality of life for both family and patient by means of relieving suffering in the prehospital environment.

*c) Home-based care.*

One practitioner mentioned the value of home-based care and how EMS could play a role in its provision. She stated that palliative treatment could be administered in the patient’s home without subsequent transport to a healthcare facility which is current practice in the South African environment. Lord et al. found that Australian paramedics also felt their requirement to transport these patients to hospital inhibited their ability to provide palliative care [12].

*“But now you uproot that entire system and process … of them being at home and wanting to die at home because … that’s how the system is designed.”* – Interview #4F.

Home-based palliative care is supported practice in South Africa however, its implementation is lacking [17]. Current EMS practice in South Africa further adds to this problem in terms of mandated transport of patients to a medical facility should treatment have been administered. The hospitalisations which result increase demand on already strained palliative care resources in the low-to-middle income (LMIC) setting of South Africa [17]. Supported palliative care practices and EMS systems are therefore in conflict. However, EMS systems could be used to enhance home-based care as EMS already work in patient homes and are equipped for prehospital healthcare.

An interviewee felt EMS could play an important role in facilitating transport of patients to their homes to die in comfort rather than in a medical facility. While the use of EMS to transport palliative patients from medical facilities to their home is not described in literature, the concept is worth researching as it could result in increased patient comfort, dignity and respect of patient and family wishes. These concepts are essential for the appropriate care of palliative patients [6, 8, 9]. However, this practice may strain EMS system resources, resulting in negative impact on service delivery to emergencies.

These suggested roles are not foreign to EMS providers [10, 11]. EMS providers are trained in decision-making processes, patient communication, handover processes, analgesic strategies and acting as mediators between the pre- and in-hospital healthcare services [10, 11]. EMS provider roles may, therefore, be well-suited to the provision of palliative care as they appear to fulfil the mandate of palliative care: to improve quality of life [1].

### Challenges to pre-hospital palliative care

When considering the implementation and practice of palliative care in the pre-hospital setting, participants identified numerous challenges which they felt needed to be addressed in order to implement prehospital palliative care programmes. Concerns were a) the current EMS mindset, b) lack of training, c) resource and scope of practice considerations, d) EMS system failures and e) difficulties specific to the pre-hospital field.

*a) EMS mindset.*

Interviewees highlighted the need for a difference in approach to patients requiring palliation when compared to those suffering an emergency. The current EMS mindset, they explained, is to intervene and get the patient to hospital alive at all costs; ‘saving lives’ being the dominant EMS ethos in South Africa as it is in the USA and Europe [5, 12]. While this may be a major role of EMS providers, they argued that in end-of-life situations where medical treatment would be futile, this mindset would be inappropriate. According to them the paradigm would need to shift from a life-saving approach to a relief of suffering approach before prehospital palliative care could be effective.

*“… there’s very much this thing of we as paramedics need to save every single life. We need to get every single patient to hospital alive … So I think it’s gonna be a big mindset change …”* – Interview #1 M.

This is common elsewhere around the world, where EMS providers are forced to abandon their training when confronted with palliative situations [4–6, 8, 12]. While EMS and palliative mindsets seem to be in conflict, it has been suggested they may be complimentary if integrated [4, 8, 21]. This appears logical based on potential EMS provider functions regarding palliative patients.

Some participants felt pre-hospital palliative care would not be well-suited to every EMS provider; only specific providers with the *“capabilities and the will”* to perform palliation would be suitable.

*“The number one criteria for me would be people that are actually passionate about it* [palliative treatment] *and interested in it.”* – Interview #4F.

This is the recommendation of Lamba, et al. who state the first step in integrating palliative care with EMS systems is identifying *“EMS-palliative care champions.”* [10].

*b) Lack of training.*

The lack of training at undergraduate level in palliative care was identified as a challenge. No participants received training on the topic until after graduation, where even then most knowledge was self-gained. Even with knowledge and experience gained over time, participants lacked confidence to practice palliative care in the pre-hospital setting. One participant stated he would be *“hesitant”* without training while others expressed their need for further education on the correct approach to palliative patients.

*“I know now that we need to do it* [palliative care]*, it can be done safely and should be done for certain patients. And now it’s just to get more training in how to do it the correct way.”* – Interview #1 M.

*“I can’t actually remember having been introduced to the idea of palliative care at all in* [undergraduate level]*.”* – Interview #3F.

Globally, EMS educational material on the subject is minimal and training has been called for in many studies [3–5, 8, 13]. This need for training is present in both physician and paramedic-based EMS systems [3, 5, 8]. A German study found that while 89% (*n* = 93) of physicians had encountered palliative care patients, only 32% (*n* = 33) were trained in palliative care [9]. While these data have not been researched in the South African setting, it is likely the need for training is greater due to the higher burdens of disease.

Two unique points regarding the need for training were raised by Interviewee #5 M. He stated that education should be aimed at all levels of EMS providers, including BLS, as ALS providers do not respond to the majority of calls. According to him, this would make prehospital provision of palliative care more *“equitable”*. Secondly, he noted the need for patient and family education. They too should receive education on palliative care to improve communication with EMS providers about the management of such patients. This is consistent with views of American and Australian paramedics who likewise found the lack of family education challenging [7, 12].

*c) Resource and scope of practice considerations.*

Interviewee #4F provided an example from personal experience of how limited scope of practice fails palliative care patients: *“… there’s someone at home on a vent and you can hear there’s a bit of extra secretions, there’s a temperature … patient probably only needs a course of antibiotics. In reality, in normal life, the mom would put her kid in the car and go to the GP and get antibiotics. Because the child is ventilated she doesn’t have that freedom of movement. Now you must arrange a whole big transfer to hospital.”* She concluded by saying, *“… that function should be available to them at home”* suggesting antibiotics be included in ALS scope of practice.

While she and other participants felt scope of practice and resources were a limiting factor to the provision of pre-hospital palliative care, others felt these were adequate.

*“… I think from a resource point of view in my current work setting there’s no reason why it [palliative care] cannot be implemented.”* – Interview #1 M.

*“… I do believe that we’re very limited from an analgesic … perspective.”* – Interview #2 M.

Apart from medications, participants did express other limiting factors regarding palliative care management. They stated the following was not currently practiced, but would be useful: on-scene discharge, prescription of medications and changing of tracheostomies.

German paramedics also have scope of practice limitations which inhibit their ability to provide appropriate care to palliative patients [2]. In an end-of-life situation which has deteriorated to cardiac arrest they are forced to initiate mechanical and pharmacological resuscitative efforts in the absence of a physician [2]. While South African ECPs have more freedom in terms of when to withhold resuscitative efforts, they still lack in terms of procedural scope. While EMS provider scopes of practice may differ around the world, they all may experience limitations when facing palliative situations.

Resource limitations are not highlighted in prehospital palliative care studies in HICs [9, 12]. However, to integrate palliative care into EMS systems it is recommended a needs assessment be performed to identify required resources [10]. This is particularly relevant in the South African LMIC setting.

*d) EMS system failures.*

Participants were concerned that when they do implement appropriate palliative care measures the system does not defend them and may discipline them. Other system problems noted were logistical difficulties (i.e. what hospital should these patients be transported to?), lack of communication amongst role players and questions surrounding documentation of palliative care management.

*“I think we have a very valuable role to play, but it’s almost being limited by how things are structured …”* – Interview #4F.

*“The family is telling me not to intubate this patient, but there was no legal document … so I was advised* [by Medical Officer] *to rather go the whole invasive treatment... So I intubated the patient and I knew that it was against her wishes and against the family’s wishes …”* – Interview #6F.

According to a study on prehospital palliative care protocols in the USA, EMS protocols are typically designed for patients requiring curative measures as opposed to comfort care [6]. This explains the dilemma highlighted by Interviewee #6F. EMS provider discomfort around deviating from system protocol is well documented [6, 12, 13]. This has been found to limit their ability to manage palliative situations [13]. Participants in this study have similar limitations. The integration of palliative care into South African EMS systems, therefore, appears warranted to improve palliative patient management.

To further illustrate the dysfunction of the current system, one practitioner described a situation where she was called to a palliative healthcare facility to resuscitate a patient in cardiac arrest. *“Isn’t that where a patient should be left to die?”* she asked. This may be due to a failure of palliative care in South Africa. A study in France noted a similar problem revealing that emergency care was sought in end-of-life situations for patients already in palliative care in nearly 30% of cases [33]. The authors labelled this a *“disturbing finding”* highlighting the potential lack of training even among palliative care teams [33].

Despite these system failures, some participants felt able to perform certain elements of palliative care, such as providing supportive care rather than full resuscitative care, in their current setting provided they were able to justify their management during clinical reviews. Others, however, feared the risk and felt unable to perform palliative care without repercussions from their employer.

*e) Difficulties specific to the pre-hospital field.*

Further identified challenges result from unique pre-hospital patient management difficulties. The interviewees described difficulties in intervention decisions, dealing with patient family members, gathering information and with personal mental health. Determining when to initiate palliative care presented the potential for harm to both the practitioners and the patient. A patient requiring resuscitative treatment for survival could be mistaken as a terminally ill patient requiring palliation resulting in adverse outcome. According to participants, making these decisions is difficult in the prehospital setting as scene-time and information are limited. Furthermore, family and patient wishes may clash with each other or with practitioner clinical reasoning.

*“… for me personally it was difficult making that decision* [palliation vs ALS resuscitation] *with 2 minutes’ worth of history on a patient you’ve never encountered and that you will never see again after the call’s over in 30 minutes.”* – Interview #1 M.

*“You might have family members that say yeah you have to do everything, even though you know this patient is more eligible for palliative care than resuscitation.”* – Interview #6F.

*“I think from … an emotional welfare point of view … you need to be comfortable then with the decision that you made.”* – Interview #3F.

The prehospital field is more challenging for healthcare providers than the controlled in-hospital setting [33, 36, 37]. Ferrand and Marty concur with the participants of this study that future quality of patient life is impossible to predict in the emergency pre-hospital setting and thus, viability decisions in palliative situations are challenging [33]. The lack of on-scene medical information available to EMS providers, particularly in palliative situations, has been highlighted as a challenge around the world [10, 13, 35]. This has been found to create conflict between EMS providers, palliative patients and patient family members as dilemmas develop over competing interests: patient wishes vs. family wishes vs. EMS protocols vs. legalities [7, 10, 35].

Advance directives (ADs) and do not attempt resuscitation orders (DNARs) can be helpful to healthcare providers regarding decision making when managing palliative care patients as they give insight into patient wishes and legalities of treatment measures [12]. However, rather than assisting EMS providers in palliative situations, these documents have been shown to cause more confusion [2–5, 7, 34]. EMS providers are often unaware whether their patients have ADs or DNARs due to lack of available information [5, 7, 10]. Furthermore, when ADs and DNARs are presented, EMS providers are unsure of their legal implications in emergency settings as there are no clear guidelines for their use [2, 12, 34]. In South Africa the same confusion exists as there is no legal guidance regarding ADs or DNARs in the pre-hospital setting [38].

### Ideas for implementing pre-hospital palliative care

Despite the challenges of pre-hospital palliative care in South Africa, participants felt implementation was still necessary and possible. Their ideas for implementation were a) the creation of guidelines, b) a multi-disciplinary approach and c) the creation of pre-hospital palliative care specialists or teams.

*a) Creation of guidelines.*

To overcome fear of practicing palliative care in their respective environments due to litigation or company repercussions, candidates stated guidelines should be developed and implemented. This would give them confidence by providing direction, system, legal and ethical backing when managing palliative patients.

*“I think anybody would feel much more comfortable making those decisions if they knew that higher-up they would be backed-up.”* – Interview #3F.

*“I think that* [policy] *will also just give the practitioner ease of mind to know they’re covered...”* – Interview #1 M.

Calls for palliative care guidelines in EMS systems have been made previously [2, 6, 10]. A recent Canadian study found the introduction of one such guideline successful in improving paramedic comfort and confidence when performing prehospital palliative care [39]. As specific guidelines on this topic do not currently exist within South African EMS structures their development would be beneficial. This will require further research on what would constitute safe and effective practice of pre-hospital palliative care.

*b) Multi-disciplinary approach.*

Along with guidelines, participants felt pre-hospital palliative care should involve a multidisciplinary approach with involvement of others. This would include family members, treating physicians and other specialists. The idea would be to ensure that correct investigations and treatment measures are performed before officially withdrawing treatment or declaring a patient for palliation. In so doing the decision-making regarding patient prognosis and management would not rest solely on the EMS providers. Thus, the risk of mistakenly performing palliation on a patient requiring more invasive treatment and vice versa would be reduced.

*“There would’ve had to be a multi-disciplinary approach with counsellors, with the treating specialist, with everyone involved, making the decision to say ok it’s end-of-life, we are going to withdraw continuous care.”* – Interview #4F.

Palliative care itself makes use of a multi-disciplinary approach [3]. Thus, the integration of palliative care into EMS would result in EMS providers becoming role players. Many studies concerning prehospital palliative care have recommended a multi-disciplinary approach to provide the best possible care for palliative patients in all environments [4, 8–10, 35]. This should be considered in the South African prehospital setting.

*c) Creation of pre-hospital palliative care specialists/teams.*

Participants mentioned the possibility of deploying specialised pre-hospital palliative care units which would *“fundamentally change the scope”* of prehospital healthcare influence. Candidates did not elaborate on what personnel the unit would consist of; however, a specialized unit would be able to incorporate not only EMS providers, but specialists from other medical disciplines, providing for a multi-disciplinary approach.

*“In my view we might have to have pre-hospital specialists who have specialized in palliative medicine … I think it would be such an important additional component.”* – Interview #2 M.

*“I literally think you could have a specialised unit dedicated to palliative care. That’s how much of a need there is for it.”* – Interview #4F.

Quality of palliative care in the prehospital setting has been found to depend on the expertise of the healthcare providers present [21]. Thus, implementing specialist prehospital palliative care teams may improve quality of palliative patient care. While the use of specialist pre-hospital palliative care teams has not been researched in South Africa or abroad, Wiese, et al. have stated the necessity of integrating these specialist teams to support both the palliative patient as well as EMS providers faced with palliative situations [3].

### Limitations

The qualitative design of the study resulted in the ability to gather the perspectives of participants however, these may not be representative of other providers within differing areas of South Africa or other countries. Furthermore, study participants all graduated from a single institution. While these limitations to transferability are apparent, the findings of this study correlate well with the findings of similar studies performed in other countries. The voluntary nature of participation in the study may mean only those with strong feelings about the topic participated resulting in possible self-selection bias. However, their opinions should be seen as no less valuable.

## Conclusion

The aim of this study was to begin exploring the topic of South African prehospital palliative care by gathering the perspectives of ALS providers within the South African private EMS sector concerning prehospital palliative care importance, feasibility and barriers to practice. Four categories arose from interview analyses: 1) need for pre-hospital palliative care, 2) function of pre-hospital healthcare providers concerning palliative care, 3) challenges to pre-hospital palliative care and 4) ideas for implementing pre-hospital palliative care. According to the interviewees of this study, pre-hospital palliative care in South Africa is needed and EMS providers can play a valuable role. However, they identified many challenges such as a lack of education and EMS system and mindset barriers. These barriers, they said, may be overcome by developing guidelines, training, and a multi-disciplinary approach. Given the paucity of research on the topic of prehospital palliative care in South Africa, the quadruple burden of disease described in literature and the personal experiences of the participants interviewed, the importance of developing prehospital palliative care within South Africa is apparent and plans for implementation should be developed.

Further research should be conducted within different South African settings, to further expand on the topic of prehospital palliative care, including the public sector which services different patient populations and may have more severe resource limitations. Research is needed to determine what effect prehospital palliative care would have on patients as well as develop guidelines and curricula for training purposes.

## Supplementary information

**Additional file 1.**

## Data Availability

The datasets used and/or analysed during the current study are available from the corresponding author on reasonable request.

## Abbreviations

AD: Advance Directive
AIDS: Acquired Immune Deficiency Syndrome
ALS: Advanced Life Support
BLS: Basic Life Support
COREQ: COnsolidated Criteria for Reporting Qualitative research
DNAR: Do Not Attempt Resuscitation
ECP: Emergency Care Practitioner
EMS: Emergency Medical Service
HE: Higher Education
HEMS: Helicopter Emergency Medical Service
HIC: High Income Country
HIV: Human Immunodeficiency Virus
HREC: Human Research Ethics Committee
ICU: Intensive Care Unit
LMIC: Low-to-Middle Income Country
RVD: Retro-Viral Disease
UCT: University of Cape Town
USA: United States of America
WHO: World Health Organization
