# Supplementary material for: South African paramedic perspectives on prehospital palliative care
Source: BMC Palliat Care. 2020 Oct 8;19:153. doi: 10.1186/s12904-020-00663-5 (PMC7545550; doi:10.1186/s12904-020-00663-5)
Supplement: Supplementary file 1 — Additional file 1. [file 12904_2020_663_MOESM1_ESM.docx]

**Interview Schedule**

THE THOUGHTS AND OPINIONS OF ADVANCED LIFE SUPPORT PROVIDERS IN THE SOUTH AFRICAN PRIVATE EMERGENCY MEDICAL SERVICES SECTOR CONCERNING PRE-HOSPITAL PALLIATIVE CARE

NOTES TO INTERVIEWER: Statements in *ITALICS* are instructions to the interviewer. Questions and text to be read out are in **BOLD.** Prompts contained in textboxes may also be read out during the interview to encourage further dialogue or where questions require additional explanation.

FACILITATION: *Before the interview, refer to these notes to ensure familiarity with the content. All interviews should be run by the primary researcher. The primary researcher is to lead the discussion and take notes as well as operate the audio recording device. Each interview should lead to new thoughts, ideas and conclusions unless it is determined that theoretical saturation has been reached.*

PREPARATION: *Audio recording equipment and its sensitivity should be tested from various positions within the room before the interview. Ensure that participant consent forms and note-taking material is ready.* *Offer participant refreshments. Ensure the participant is as comfortable as possible before beginning the interview. Confirm that the participant has signed the consent form and that they consent to being audio recorded.*

CONFIDENTIALITY STATEMENT: **There are no right or wrong answers or opinions on the topic we will be discussing. We are here to gather your own personal and valuable thoughts. All your views, thoughts, opinions and answers will be kept confidential. I would also like to remind you that your consent can be withdrawn at any time, including during this process.** *(Give opportunity for questions on confidentiality)*

*SESSION INTRODUCTION*: *Start by briefly introducing yourself and the interview. Ask the participant to introduce themselves. You may start the session as follows:*

**Identify participant on the recording by study number and gender**

**Thank you for taking the time to discuss the topic of pre-hospital, palliative, end-of-life care in South Africa. In essence, palliative care refers to the specialized treatment of terminally ill patients. Treatment is aimed at improving quality of life as well as relieving suffering. The purpose of this interview is to gather your thoughts and opinions on the importance of this care, concerns to performing this care and feasibility of performing this care specifically in the South African private pre-hospital setting.**

**I will start by briefly asking about yourself and your experience. I will then ask three questions on the topic which we can discuss. If, at any point, you have any questions or need clarification please feel free to ask whenever you would like.**

**Could you please begin by telling me about your career and background?**

| *PROBES AND PROMPTS* |
| --- |
| *Demographics: Age, Gender, Qualification, Position.*  *Years of experience.*  *Previous experience/positions.* |

**How important would you say the practice of palliative care is in the pre-hospital setting?**

| *PROBES AND PROMPTS* |
| --- |
| *Optional vs. Necessary.*  *Patient suffering (i.e. pain, dyspnoea, anxiety).*  *Opportunity to practice this care.*  *Number of patients requiring this care.* |

**Would you have any personal concerns with performing palliative care?**

| *PROBES AND PROMPTS* |
| --- |
| *Legal concerns: current policies, fear of litigation, euthanasia, possibility of causing further harm, misdiagnosing end-of-life situation*  *Ethical concerns: EMS goal of saving life, euthanasia, hastening death, conscience*  *Technical concerns: lack of training/education, scope of practice*  *Note: Several of these may lead naturally into the next question.* |

**What do you think about the feasibility of palliative care in the specific South African private pre-hospital setting(s) which you are or have been in?**

| *PROBES AND PROMPTS* |
| --- |
| *Resources: medications, procedural equipment*  *Finances*  *Lack of qualified personnel*  *Scope of practice*  *Lack of training/education* |

SESSION CONCLUSION: *Once all the questions have been asked and answered you may conclude the session as follows:*

**Thank you again for spending your time on discussing this topic and taking part in this study. Your thoughts and opinions are very valuable and I appreciate your contribution.**
